# Just the facts: tumour lysis syndrome

**DOI:** 10.1007/s43678-026-01197-0

**Published:** 2026-06-21

**Authors:** Brit Long, Arleigh McCurdy, Hans Rosenberg

**Affiliations:** 1 Department of Emergency Medicine, University of Virginia Medical Center, Charlottesville, VA USA; 2 Division of Hematology, Department of Medicine, University of Ottawa, Ottawa, ON Canada; 3 Department of Emergency Medicine, University of Ottawa, Ottawa, ON Canada

**Keywords:** Tumour lysis syndrome, Just the facts, Oncology, Hemaetology, Syndrome de lyse tumorale, Les faits, Oncologie, Hématologie

## Case scenario

A 23-year-old male with Burkitt lymphoma, a highly proliferative B-cell malignancy, presents with fatigue, nausea, vomiting, myalgias, and decreased urine output. He started chemotherapy 2 days ago. He appears fatigued, dehydrated, and is tachycardic. The rest of his vital signs are within normal limits.

### What is tumour lysis syndrome and its pathophysiology?

Tumour lysis syndrome is an oncological and metabolic emergency that most commonly occurs in malignancies with high proliferation and cell turnover, associated with extensive tumour cell death with release of nucleic acids, uric acid, phosphate, potassium, cytokines, and interleukins [1–6]. Due to significant electrolyte abnormalities (especially hyperkalaemia and hypocalcaemia) patients are at risk of end organ dysfunction involving the neurological, renal, and cardiac systems. Release of uric acid results in oxidative stress, inflammation, and end organ damage. The release of cytokines and interleukins may lead to severe systemic inflammation, hypotension, and organ failure [4–6].

Tumour lysis syndrome diagnosis is based on the Cairo-Bishop definition, comprising laboratory and clinical criteria (Table 1) [2]. To meet laboratory criteria for diagnosis, patients must have two or more laboratory findings. For clinical tumour lysis syndrome, patients must meet the laboratory criteria and have at least one clinical finding. In-hospital mortality approximates 21%, and up to 69% of patients experience at least one major complication [7].

**Table 1:** Cairo-Bishop definitions of tumour lysis syndrome

Laboratory tumour lysis syndrome	Clinical tumour lysis syndrome
*2 or more of the following*	*Laboratory Tumour Lysis Syndrome plus one of the following:*
● Uric acid	● Creatinine ≥ 1.5 × greater than upper limit of normal
○ ≥ 0.48 mmol/L OR	● Cardiac arrhythmia/sudden death
○ 25% increase from baseline	● Seizure
● Potassium	
○ ≥ 6.0 mmol/L OR	
○ 25% increase from baseline	
● Phosphorus	
○ ≥ 2.1 mmol/L (children), ≥ 1.45 mmol/L (adults) OR	
○ 25% increase from baseline	
● Calcium	
○ Corrected calcium < 1.75 mmol/L, ionized calcium < 0.28m mol/L OR	
○ 25% decrease from baseline	

### What factors are associated with tumour lysis syndrome?

**Fig. 1:**
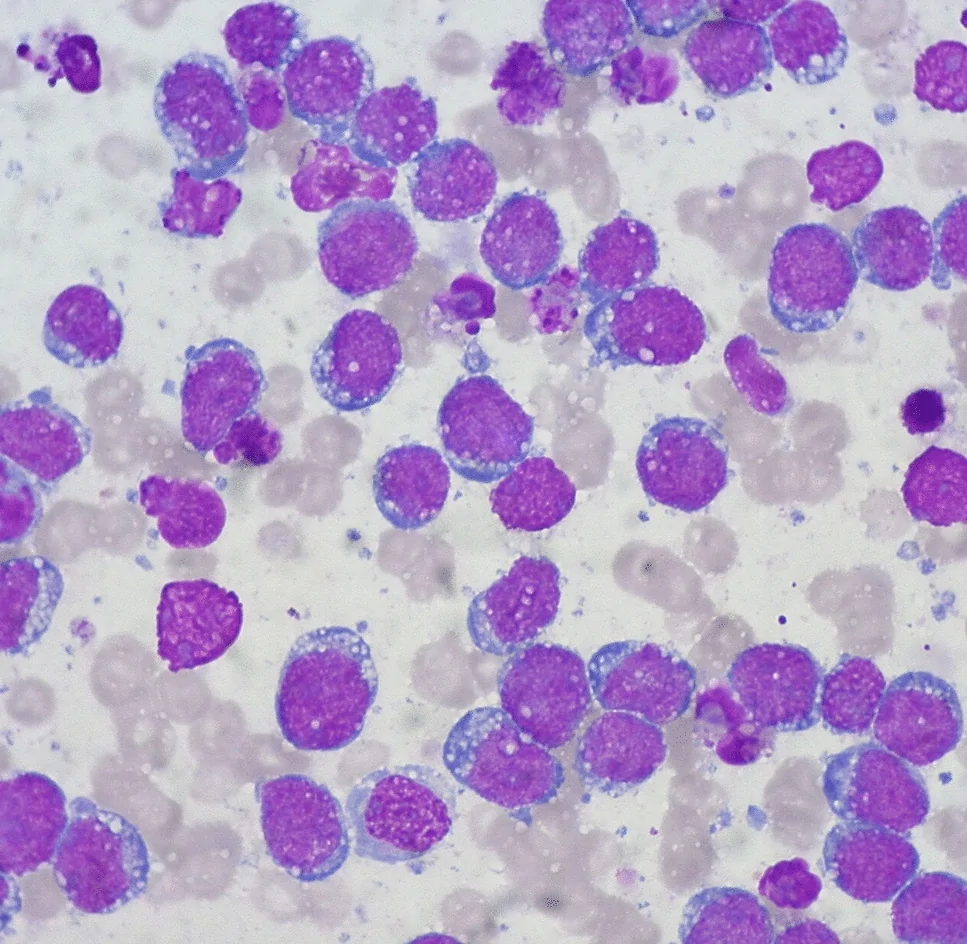
Burkitt lymphoma histopathologic slide with Giemsa stain. Obtained from https://commons.wikimedia.org/wiki/File:Burkitt_Lymphoma_%28FNA_Giemsa_stain%29_%284679964756%29.jpg

The incidence of tumour lysis syndrome ranges between 4 and 42% and depends on the underlying cancer type and patient characteristics [8, 9]. Cancers with significant risk of tumour lysis syndrome include non-Hodgkin lymphoma (30%), solid tumours (20%), acute myeloid leukaemia (19%), and acute lymphocytic leukaemia. Patients with Burkitt lymphoma are susceptible to tumour lysis syndrome due to the malignancy’s rapid cell turnover, significant tumour burden, and high sensitivity to chemotherapy (Fig. 1). Large tumour burden (white blood cell count > 15,000/mm^3^, bulky disease > 10 cm, bone marrow involvement, splenomegaly/hepatomegaly) and renal infiltration or outflow obstruction also increase the risk [7–9]. Patient-related factors include hypovolaemia, hypotension, pre-existing kidney disease, high uric acid/phosphate prior to treatment, and elevated lactate dehydrogenase. Treatment-related risks include chemotherapy intensity, combination therapy, and nephrotoxin use [4–6].

Tumour lysis syndrome is associated with several triggers, but it most commonly occurs within 72 h of cytotoxic chemotherapy [6, 9]. It may also occur with radiation, embolization, monoclonal antibody infusion, chimeric antigen receptor-T cell therapy, kinase inhibitors, proteasome inhibitors, thalidomide/lenalidomide, venetoclax, and hydroxyurea. Rarely, it can occur spontaneously in aggressive tumours even prior to treatment.

### How does tumour lysis syndrome present in the emergency department?

Acute kidney injury may present with oliguria/anuria, fatigue, dysuria, haematuria, and flank pain. Severe hyperphosphataemia may lead to lethargy, nausea, vomiting, diarrhoea, and seizures. Hypocalcaemia is associated with muscle cramps, tetany, paresthesias, renal injury, and seizures. Hypocalcaemia and hyperkalaemia may result in cardiac dysrhythmias. Patients with uraemia may be altered and display pruritus, hiccups, restless legs, dysgeusia, and bruising. Cardiopulmonary complications include dysrhythmias, syncope, heart failure, and volume overload [1–6]. Due to the varied presentation and range of symptoms of tumour lysis syndrome, an extensive laboratory evaluation is recommended in patients receiving acute therapy for pertinent malignancies (Table 2).

**Table 2:** Emergency department evaluation for suspected tumour lysis syndrome

Recommend for all patients:	Based on clinical picture:
● Complete blood count	● Acute coronary syndrome—troponin
● Electrolytes	● Heart failure—brain natriuretic peptide
● Renal/liver function	● Infection—blood cultures, urinalysis, chest X-ray
● Uric acid level	
● Calcium level	
● Magnesium level	
● Phosphate level	
● Lactate dehydrogenase	
● Urinalysis	
● Electrocardiogram	

### What are the red flags and what are common pitfalls in the evaluation of those with suspected tumour lysis syndrome?

In patients with cancer undergoing chemotherapy, laboratory testing revealing acute kidney injury, elevated uric acid, hyperphosphataemia, hyperkalaemia, and/or hypocalcaemia suggest tumour lysis syndrome [1, 2, 4, 5, 8]. Electrocardiogram may reveal evidence of conduction blocks or hyperkalaemia (peaked T waves, prolonged PR interval, junctional rhythm, QRS prolongation, sinusoidal wave pattern) [6]. Lactate dehydrogenase is usually elevated and reflects rapid cell turnover [8]. If one of more of the laboratory findings is present, clinicians should review the Cairo-Bishop criteria [2].

#### Differential diagnosis

Due to the non-specific presentation with fatigue, weakness, or signs of dehydration, emergency clinicians should consider other diagnoses including hypovolaemia secondary to decreased oral intake or gastrointestinal losses, infections (bacterial, viral, fungal), neurological complications of haematological malignancies (e.g. leptomeningeal metastases, spinal cord compression, paraneoplastic syndromes), and chemotherapeutic agent side effects.

### What are the primary medical therapies for tumour lysis syndrome?

**Fig. 2:**
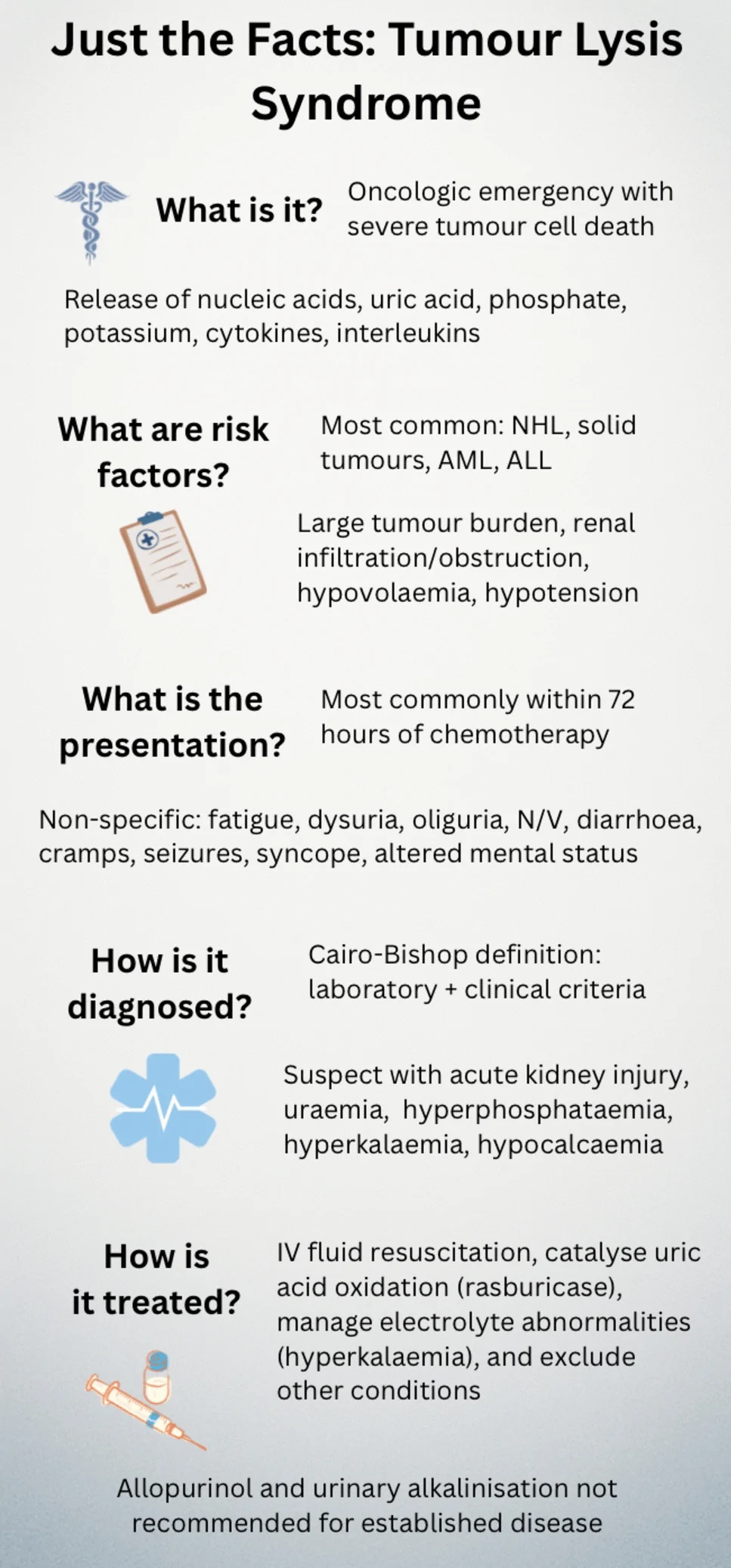
Tumour lysis syndrome infographic

Primary treatment goals include intravenous fluid resuscitation, catalysing uric acid oxidation, managing electrolyte abnormalities, and excluding other dangerous conditions (Fig. 2) [1–5]. Consultation with the haematology/oncology and nephrology specialists is necessary, and treatment may warrant transfer to a tertiary care centre. Fluid resuscitation should target euvolaemia, followed by administering intravenous fluids at 150–200 mL/h and targeting a urine output of 80–100 mL/h [10]. Whilst fluids with calcium, phosphate, and potassium are generally avoided, this recommendation is based on expert consensus, rather than high-quality data. Rasburicase oxidises uric acid to allantoin and is recommended in those with uric acid levels over 75 mg/L. Literature suggests adult dosing can range between 3 and 6 mg intravenous [11]. Whilst rasburicase is generally well tolerated, there is a risk of anaphylaxis. It is contraindicated during pregnancy, during lactation, and in patients with glucose-6-phosphate dehydrogenase deficiency [8]. Patients with hyperkalaemic electrocardiogram changes should be treated with intravenous calcium and standard therapies to shift and excrete potassium. Furosemide is recommended in those with low urinary output despite hydration or volume overload [10]. Thiazide diuretics should be avoided, as they increase serum uric acid levels. Hyperphosphataemia is treated with intravenous fluid resuscitation. Hemodialysis indications include oliguria or anuria; symptomatic hypocalcaemia unresponsive to supplementation (corrected calcium < 1.75 mmol/L, ionized calcium < 0.3 mmol/L); volume overload not amenable to diuresis; symptomatic uraemia; refractory acidosis; and refractory hyperkalaemia, hyperphosphataemia, or hyperuricaemia [4, 8].

Allopurinol is used for prevention of tumour lysis syndrome; however, it is not recommended for patients with established tumour lysis syndrome unless there is a contraindication to rasburicase [4–6].

Urinary alkalinisation with sodium bicarbonate was historically recommended for tumour lysis syndrome treatment; however, it can promote calcium phosphate precipitation, further decreasing serum calcium, and should not be performed [8, 10].

## Case conclusion

Laboratory testing reveals elevated serum creatinine, potassium, phosphate, and uric acid and low calcium. The clinician starts intravenous fluid resuscitation and administers ondansetron. Concerned for tumour lysis syndrome, they consult the haematologist, who recommends transfer to the tertiary care centre for rasburicase and admission with further monitoring.

## Data Availability

We have no data we used for the construction of this manuscript.
